# Strategies to Overcome Enrollment Barriers

**DOI:** 10.1093/geroni/igaa057.1757

**Published:** 2020-12-16

**Authors:** Phyllis Cummins

**Affiliations:** Miami University, Oxford, Ohio, United States

## Abstract

The importance of postsecondary education for economic vitality and individual opportunity has received increased focus with over 40 states embracing postsecondary attainment goals for their populations in alignment with Lumina Foundation’s goal that 60% of individuals ages 25-64 have a recognized credential by 2025. Credentials that both meet the needs of an aging society and move the country towards achieving attainment goals are widely available at community colleges. Community colleges are not only important sources of training and education for adult learners, they also work closely with employers to meet their workforce needs. However, adult learners face many barriers to college enrollment, including poor pre-enrollment advising and lack of understanding of financial aid options. Examples of successful strategies to facilitate enrollment will be discussed, including navigators who serve as holistic advisors and work with students from pre-enrollment to graduation, and also guide the prospective student through the financial aid process. Part of a symposium sponsored by the Community College Interest Group.

